# Gastro-Esophageal Reflux in Children

**DOI:** 10.3390/ijms18081671

**Published:** 2017-08-01

**Authors:** Anna Rybak, Marcella Pesce, Nikhil Thapar, Osvaldo Borrelli

**Affiliations:** 1 Department of Gastroenterology, Division of Neurogastroenterology and Motility, Great Ormond Street Hospital, London WC1N 3JH, UK; anna.rybak@gosh.nhs.uk (A.R.); marcella.pesce@gosh.nhs.uk (M.P.); n.thapar@ucl.ac.uk (N.T.); 2 Department of Clinical Medicine and Surgery, University of Naples Federico II, 80138 Napoli, Italy; 3 Stem Cells and Regenerative Medicine, UCL Institute of Child Health, 30 Guilford Street, London WC1N 1EH, UK

**Keywords:** gastro-esophageal reflux, children, infants, extraintestinal symptoms, guidelines

## Abstract

Gastro-esophageal reflux (GER) is common in infants and children and has a varied clinical presentation: from infants with innocent regurgitation to infants and children with severe esophageal and extra-esophageal complications that define pathological gastro-esophageal reflux disease (GERD). Although the pathophysiology is similar to that of adults, symptoms of GERD in infants and children are often distinct from classic ones such as heartburn. The passage of gastric contents into the esophagus is a normal phenomenon occurring many times a day both in adults and children, but, in infants, several factors contribute to exacerbate this phenomenon, including a liquid milk-based diet, recumbent position and both structural and functional immaturity of the gastro-esophageal junction. This article focuses on the presentation, diagnosis and treatment of GERD that occurs in infants and children, based on available and current guidelines.

## 1. Introduction

Gastro-esophageal reflux (GER) refers to the involuntary passage of gastric contents into the esophagus. In children, it often represents a physiological phenomenon, especially in infants with innocent regurgitation. Conversely, GER disease (GERD) occurs when the reflux of gastric contents causes troublesome symptoms and/or complications. It is one of the most common causes of foregut symptoms across all pediatric age groups [1,2].

Even though the pathophysiology and symptoms, especially in older children, of pediatric GERD are similar to those in adults, children may also present with a wide range of distinct gastro-esophageal and extra-esophageal symptoms and potential complications [3].

The conservative approach of “educate-test-treat” seems to be especially important in infants, where regurgitation most commonly reflects physiological immaturity of the gastro-esophageal junction, including a short distance and lack of the acute angle between the esophagus and the gastric fundus (angle of His), where the food is initially stored after ingestion. This physiological immaturity is often transient and improves without any medical intervention but raises the possibility of over-medicalization of GER during infancy and potential adverse consequences of attempted treatment without expected clinical benefits. There are, however, pediatric patient with potentially severe or persistent GERD, which warrant further assessment and attention given to the potential long-term consequences of the disease itself, with consideration to side effects of its treatment as well. The following article focuses on the presentation, diagnosis and treatment of GERD in the pediatric population as well as differences with GERD in adults.

## 2. Evolution and Natural Course of Regurgitation in the Pediatric Age Group

The passage of gastric content into the esophagus (i.e., GER) is a normal phenomenon occurring many times a day, in both adults and children. Infants are especially prone to regurgitate and it has been shown that the number of infants with this phenomenon decreases from about 80% during the first month of life to less than 10% at the age of one year [4]. A study by Miyazawa et al. on 921 infants showed that over 47% of one-month-old infants have one or more regurgitation or vomiting episodes per day, however this number falls to just 6.4% by the age of seven months [5]. Several factors contribute to exacerbate this phenomenon in the youngest infants, including the sole or predominantly liquid milk-based diet, the recumbent position and the immaturity of the function and structure of the gastro-esophageal junction [6].

Understanding the natural history and outcomes of GERD in children is very important to identify patients at risk of GERD-related complications and the persistence of symptoms into the adulthood. In a longitudinal study, Orenstein et al. studied infants with GERD symptoms and histological changes in the esophagus in the first year of life and found that in a significant proportion of infants histology remained abnormal, despite complete resolution of symptoms without pharmacotherapy [7]. In a retrospective/prospective cohort study of adolescents and young adults who were diagnosed in childhood with GERD, defined by erosive esophagitis, it was shown that later in life almost 80% of these patients had at least monthly heartburn and/or acid regurgitation and almost one third was taking either anti-secretory drugs or proton pump inhibitors [8]. In another study by Waring et al., 255 adult patients with GERD and 154 “non-refluxers” were given questionnaires asking them to recall childhood symptoms of GERD. The study showed that adults with GERD were more likely to have experienced GER symptoms during childhood [9]. These studies suggest that in a significant percentage of children with GERD, symptoms may persist throughout the adolescence until the adulthood. However, large longitudinal studies are still needed to prove this relation.

## 3. Clinical Presentation of GERD in the Pediatric Population

### 3.1. When to Suspect GERD?

Physiological gastro-esophageal reflux (GER) occurs in 40% to 65% of all otherwise healthy infants between the ages of one and four months making it a fairly characteristic condition of early postnatal life. Because of high rate of GER in infancy, it is important to distinguish between what is physiologic and what is a pathologic reaction or symptom. In physiologic regurgitation (“spitting” or posseting) the process is mostly passive or effortless and the endpoint of the gastric material that has refluxed up into the esophagus is commonly the oropharynx. In vomiting, the material is forcefully expelled from the mouth, however both these symptoms are sometimes difficult to differentiate, hence other symptoms or complications should be investigated [10].

GERD occurs when reflux of the gastric contents causes symptoms that are troublesome, affect the quality of life or cause pathologic complications, the details are listed below:

Weight loss or inadequate weight gain
Crying and fussiness during and after feeding
Emesis and/or hematemesis
Irritability
Anemia
Bad breath, gagging or choking at the end of feeding
Sleeping disturbance and frequent night waking
Abdominal pain
Dental erosion
Dystonic neck posturing (Sandifer syndrome)
Dysphagia
Apnea
Respiratory symptoms (aspiration, recurrent pneumonia, chronic stridor, wheezing)

These symptoms are often non-specific and can either mimic or be caused by other infancy-related conditions, such as cow’s milk protein allergy, pyloric stenosis, malrotation, overfeeding, tracheo-esophageal fistula or constipation. Therefore, a thorough medical history and examination is crucial for appropriate diagnosis and treatment.

Unlike adults and older children, infants and small children are not able to verbalize their symptoms and a number of non-verbal symptoms and signs have been used as surrogates. Irritability coupled with back arching in infants is thought to be an equivalent of heartburn in older children. Other causes of irritability and abnormal movements, including cow’s milk protein allergy, neurologic disorders, constipation and infection, should be ruled out. In children aged 2–12 years, the main symptoms include regurgitation, vomiting, abdominal pain and feeding difficulties, but typical GER symptoms can be reliably assessed in children 8–12 years of age [11]. Nelson et al. have shown how the GERD symptoms can vary depending on the age of the patient (Table 1) [3].

In their study, parents of 3- to 9-year-old children reported that their children most often experienced epigastric pain. Older children complained more of heartburn and regurgitation; however, complaints of abdominal pain were common in both age groups [3].

Certain conditions exist, which predispose to severe, chronic GERD. These include neurologic impairment, obesity, anatomical anomalies like esophageal atresia, hiatal hernia or achalasia, cystic fibrosis, lung transplantation, and a family history of GERD, Barrett’s esophagus or esophageal adenocarcinoma [11].

### 3.2. GERD and Food Allergy

In children, the prevalence of food allergy is estimated to be approximately 6–8%, with some studies reporting prevalence even of up to 18% [13,14]. Both, regurgitation and vomiting are well-recognized clinical manifestations of food allergy, mainly of cow’s milk protein allergy (CMPA), which represents the most common food allergy in early childhood. Although it is difficult to discriminate between GERD and allergy driven GER symptoms based only on clinical picture, this is particularly important with regards to the future treatment.

Although the presentation of CMPA overlaps with GERD, and both conditions may commonly coexist in both infants and children, studies addressing the relationship between these two entities showed an overall association ranging between 16% and 55%, far beyond what can be expected from pure coincidence. Nielsen et al showed that 56% of children with severe GERD were found to have CMPA on double-blind or open challenge [15]. Subsequently, Yukselen and Celtik studied the frequency of food allergy in children below five years of age with poor response of GERD symptoms to pharmacological treatment and found a prevalence of food allergy of 43% in the study group, with more than a third of patients (38.4%) showing an allergic reaction only to cow’s milk protein [16]. Previous studies on food allergy (CMPA and immunoglobulin E-dependent allergy) in GERD in children have shown similar results reporting an association between 43% and 48% [17,18].

As the prevalence of both GERD and food allergy diagnoses have increased over the last decade, together with an increase in proton-pump inhibitor and H_2_ receptor antagonist prescriptions, this has raised questions about the possible effects of altered gastric pH on the development of food allergy. Based on animal studies, antacid medication impairs the gastric digestion of proteins, with the potential of forming novel dietary proteins, which in turn could promote specific IgE synthesis and lead to food allergy [19]. Trikha et al. showed that children exposed to gastric acid suppressants due to GERD were twice as likely to be diagnosed with food allergy after a year of treatment compared to healthy controls, as well as to children with GERD on conservative (non-pharmacological) treatment only [20].

Several studies have also shown that at least in a subset of patients, GERD is not only associated with CMPA, but also can be induced by it. Indeed, Borrelli et al. showed in 17 children with CMPA and suspected GERD that cow’s milk exposure increases the number of weakly acidic reflux episodes [21]. However, taking into account that food allergy with predominant gastro-intestinal symptoms is mostly non-IgE related, other pathophysiologic mechanisms underlying the relationship between allergy and GERD should be taken into consideration and need further investigations.

### 3.3. GERD and Respiratory Symptoms

Symptoms arising from the respiratory tract are common in the pediatric population. Several studies have suggested a link between GERD and respiratory symptoms with a number of pathophysiologic mechanisms proposed to explain this, including aspiration of gastric contents into the respiratory tree, vagal reflexes induced by the presence of gastric contents in the esophageal lumen, and sensitization of the central cough reflex [22,23]. Borrelli et al compared the type and physical characteristics of reflux episodes in 24 children with cough-related GER with that found in children with erosive GERD [24]. No differences between the two groups were found in terms of total reflux episodes, number of acid, weakly acidic and weakly alkaline reflux episodes or indeed of the proximal extent of reflux episodes. They did show, however, that 66% of cough bursts were related to acid reflux episodes, whilst the remaining one third of episodes were related to either weakly acid or alkaline reflux, suggesting that cough symptoms can be associated with all types of reflux, although acid reflux appears the main determinant in the genesis of cough-related GER. Different results were shown in a study of 145 children by Zenzeri et al. Although the authors showed similar numbers of proximal reflux episodes (i.e., reflux events reaching one or two most proximal impedance channels) in patients with GERD-related respiratory symptoms compared to children with GERD presenting with only gastro-intestinal (GI) symptoms [25], significantly higher numbers of weakly alkaline reflux in the study group (children 1 year of age with reflux-related respiratory symptoms) rather than acid reflux were seen. This supported the hypothesis that reflux acidity is not the main cause of respiratory symptoms and therefore the treatment based on acid suppressants is less effective in this group of patients [25].

In a large retrospective cross-sectional study of 1980 children with GERD and 7920 controls, the authors showed a significantly higher occurrence of sinusitis, laryngitis, asthma, pneumonia and bronchiectasis in patients suffering from GERD [26]. In another study, although there appeared to be a significantly higher prevalence of asthma in children with GERD presenting with respiratory symptoms compared to subjects presenting with GI symptoms only (35.3% vs. 5.3%, respectively), the overall prevalence of asthma in patients with and without GERD was similar [27]. Therefore, although an association between asthma and GERD is advocated, the cause–effect relationship needs further elucidation.

The range of different diagnostic methodologies used in research studies affects result interpretation, including the lack of a standardized definition for respiratory disease and/or symptoms, or the lack of a clearly temporal relationship between the onset of respiratory and GERD symptoms and/or signs. Moreover, it is difficult to evaluate whether children with GERD are at increased risk of respiratory diseases in studies that do not assess the prevalence of the same disorders in a control group. Another confounding factor is that the evaluation of GERD prevalence in children with respiratory disorders by using diagnostic methodologies cannot be extrapolated to the general population, as pediatric gastroenterologists generally investigate children only after the failure of conventional therapy.

### 3.4. GERD and Extraintestinal Symptoms

Previous data suggest that younger patients tend to have more extraintestinal symptoms, whereas adolescent children more often have classical GERD symptoms [26]. Outside the respiratory tract, children can present with neurological signs of reflux like dystonia, Sandifer syndrome (i.e., torticollis of the head and neck with dystonic posturing of the upper body), opisthotonus or tic disorder, mimicking neurological disorders. In a group of 46 children with such symptoms (dystonia, Sandifer syndrome, opisthotonus, tic disorder), Pilic et al. found pathologic results of multichannel intraluminal pH-impedance in 50% of patients [28].

Ghaem at al. showed that infants with GERD (*n* = 76) had a higher prevalence of sleep disturbances requiring parental interventions and significantly delayed onset of sleeping through the night compared to non-GERD controls [29]. In some groups of patients with complex underlying conditions, e.g., neurologically impaired, syndromic, etc., GERD may be suspected in the presence of atypical or unusual symptoms. In patients with Cornelia de Lange syndrome, where reflux is a common and severe complication, a strong correlation between GERD and bruxism (i.e., grinding the teeth and clenching the jaw), nocturnal agitation and hyperactivity was suggested [30]. In older children with cerebral palsy, one of the sings of GERD is dental erosion due to the increased exposure to gastric acidic content. Other conditions related to high risk of GERD complications are listed below:

Neurologic impairment
Obesity
History of esophageal atresia (repaired)
Hiatal hernia
Achalasia (post treatment)
Chronic respiratory disorders
Bronchopulmonary dysplasia
Idiopathic interstitial fibrosis
Cystic fibrosis
History of lung transplantation
Prematurity

### 3.5. GERD and Congenital Gastrointestinal Disorders

Acquired (secondary) GERD can also occur with a number of congenital anomalies, including congenital diaphragmatic hernia, absence of diaphragmatic crura, omphalocele, gastroschisis, esophageal atresia and intestinal malrotation, with reported incidences as high as 50–84% [31]. The exact cause for GERD in some of these congenital conditions remains unknown, but it is likely to include increased intra-abdominal pressure, disturbances of esophageal motor and gastro-esophageal junction activity along with impacts from other associated anomalies, and disturbances of small bowel motor activity. With regards to diaphragmatic hernia, GERD is not only a very common concomitant disorder, but it also associated with long-term severe complications in adulthood, including Barrett’s esophagus in over 50% of cases [32,33]. Weakness of the crura and the location of the crural diaphragm (relative to sphincter location), esophageal dysmotility and shortening of the esophagus are some of the potential causes of the GERD in these patients.

The most common congenital esophageal malformation is esophageal atresia (EA), with the reported incidence ranging from one in 2500 to one in 3500 live births [34,35]. The outcome of the surgical repair depends mainly on the type of the EA, with up to 50% of those with an associated tracheo-esophageal fistula suffering from GERD [31]. This tendency seems to increase with time [36,37]. Patients with EA are more likely to develop severe GERD due to multiple reasons: impaired anatomy with hiatus hernia or abnormal position of the intrathoracic part of esophagus, as well as due to complications arising from the vagal nerve injury (gastroparesis or delayed gastric emptying and esophageal motor activity dysfunction affecting esophageal acid clearance) [38].

Further details of the underlying pathophysiologic mechanisms of GERD development go beyond the scope of this article; however, it is important to remember that, in these conditions, their management has to be weighed against the potentially long term and often severe consequences of GERD.

## 4. Current Diagnostics Guidelines

According to current guidelines [11,39,40], no single test is sufficient to make a reliable diagnosis of GERD, as it is often a result of combined clinical assessment and diagnostic tests. Nonetheless, due to the multifaceted clinical presentation and the frequent occurrence of episodic regurgitation in otherwise healthy children, discriminating what is “physiologic” gastro-esophageal reflux (GER) from what is “pathological” gastro-esophageal reflux disease (GERD) can be challenging, particularly in infants. However, the accurate distinction between these two entities is the pivotal step in the correct management of GERD, as it reflects decisions about further investigation and treatment.

Recognized GERD-promoting conditions highlighted by the most recent European and North American Society for Pediatric Gastroenterology, Hepatology, and Nutrition (ESPGHAN and NASPGHAN) guidelines are listed in the paragraph 3.4 [11]. In these populations, the likelihood of severe GERD is much higher and can predict a worse outcome of the disorder, defining an “at risk” group of patients in which further investigation and management is advisable.

### 4.1. Symptom Questionnaires

Age-specific symptom-assessing questionnaires have been developed to ease the clinical diagnosis of GERD in both, adults and children [41]. However, to date, no single symptom or cluster of symptoms has been shown to reliably identify patients with GERD and/or predict the response to treatment. The lack of correlation between reported symptoms and objectively assessed reflux relies on a number of factors and is particularly evident in infants. In this setting, GERD tends to present with a plethora of symptoms, not clearly attributable to reflux and that can mimic other conditions, particularly cow’s milk protein allergy (CMPA). Furthermore, the frequent occurrence of physiological GER and parental anxiety might lead to an overestimation of the presence of GERD in this population [42]. Consequently, clinical assessment in children aged 2 years should focus on excluding other potential worrying conditions that can present with regurgitation and vomiting, rather than making a clinical diagnosis of GERD. For this purpose, the current guidelines recognize a cluster of “red flags” symptoms that require further diagnostic testing, the details are listed below:

Bilious vomiting
Gastrointestinal bleeding
Hematemesis
Hematochezia
Consistently forceful vomiting
Onset of vomiting after 6 months of life
Failure to thrive
Diarrhea
Constipation
Fever
Lethargy
Hepatosplenomegaly
Bulging fontanelle
Macro/microcephaly
Seizures
Abdominal tenderness or distension
Documented or suspected genetic/metabolic syndrome

On the contrary, older children and adolescents tend to resemble their adult counterparts, complaining of more classical symptoms of heartburn and acid regurgitation [11], making a clinical diagnosis of GERD more consistent, in terms of specificity and sensitivity. However, the reported specificity and sensitivity of symptoms-based questionnaires varies widely and is estimated to be 70% and 65%, respectively, in adult patients with reflux disease [43]. In patients with extra-esophageal manifestations, these ratios drop further, making it very unlikely to achieve, using questionnaires, a clinical-based diagnosis of GERD in adults or children.

### 4.2. Proton Pump Inhibitors Test

The proton pump inhibitor (PPI) trial is a valuable diagnostic and therapeutic tool for GERD. It consists of an empirical short period (usually 2–4 weeks) of acid suppression with PPI therapy that can be prolonged up to 12 weeks in case of clinical improvement. The reduction by at least 50% of symptom severity after treatment is considered highly suggestive of GERD. Although biased by poor specificity [44], the PPI trial still represents the first-line therapeutic and diagnostic tool in adults, in primary care settings. Such an approach can be used in older children and adolescents presenting with typical GERD symptoms, without alarm signs. However, to date, no solid evidence supports this trial in younger children and infants; hence, although it is still a matter of huge debate, the authors discourage the “ex iuvantibus” diagnosis of GERD in these age groups, as it can lead to inappropriate prescription of PPIs and exposure to potential side effects [45,46,47]. As more extensively discussed below (see Section 5.2), the inappropriate use of acid suppressive drugs has been indeed associated with consistent modifications in the intestinal microbiota by inducing gastric hypochlorhydria, delaying gastric emptying and increasing gastric mucous viscosity [48]. In adults, chronic acid suppression has been linked to an increased risk of small intestine bacterial overgrowth (SIBO). Although not reaching statistical significance, a trend towards an increased risk of SIBO has also been recently observed in children under long-term PPIs therapy (6 months) [49]. Apart from SIBO, the chronic use of acid suppressive agents is a well-known risk factor for gastrointestinal (acute gastroenteritis, Clostidium difficile infection, candidemia and necrotizing enterocolitis) and extra-intestinal (lower respiratory tract infections, community acquired pneumonia) infections, particularly in infants.

### 4.3. Endoscopy and Esophageal Biopsies

Similar to adults, the use of an upper gastro-intestinal endoscopy in children should be reserved to patients with alarm or refractory symptoms of GERD, to excluding its complications or aimed at ruling out other conditions mimicking GERD [11]. The avoidance of unnecessary invasive testing is particularly valid in pediatric populations where the benefits of the procedure must be weighed against the possible risks of the anesthesia.

In children, the macroscopic appearance of the mucosa does not correlate with the histological findings of esophagitis. Histological examination is essential in differentiating “true” GERD from other conditions that can resemble GERD, particularly eosinophilic esophagitis (EoE), where obtaining multiple orientated biopsy samples from different areas of the esophageal body is mandatory, according to current guidelines [50]. Notwithstanding, esophageal eosinophilia is found in cow’s milk protein allergy (CMPA) patients and even in asymptomatic infants (aged 1 year) [51]. Furthermore, inflammatory infiltration of the esophageal mucosa can be patchy in children and is far more frequently seen, as compared to adults [52]. Presently, insufficient data exist to support the use of histology as a diagnostic tool for GERD in children [11,53].

### 4.4. Upper Gastrointestinal Contrast Studies

Current guidelines do not recommend routinely performing an upper gastrointestinal contrast study in the evaluation of children with suspected GERD. In selected patients, barium contrast studies may have a role to exclude conditions that may resemble GERD, such as achalasia, while, in infants, the barium meal can be useful for the diagnostic evaluation of malrotation, duodenal web and pyloric stenosis.

In addition, although endoscopy is a valid tool to exclude strictures and may raise suspicion of achalasia and malrotation, the features of the narrowing are more consistently studied with barium contrast swallows, which are less invasive. Anatomic and functional studies of the esophagus represent the gold standard in the diagnosis of these conditions.

Gastro-esophageal scintigraphy by measuring post-prandial reflux and gastric emptying has been advocated as a diagnostic tool of GERD in pediatric population; however, the lack of standardized normal values and acquisition techniques does not support its use in the diagnostic workup of GERD patients.

### 4.5. Reflux Monitoring

Both pH-metry and multiple intraluminal impedance (MII) pH-impedance monitoring are currently performed in clinical settings to evaluate the presence of reflux and the association between GER and symptoms. Despite the higher costs, the diagnostic yield of combined pH-impedance monitoring over pH-metry alone justifies the use of combined pH/MII as the test of choice in detecting GERD in children [54].

MII pH-impedance monitoring helps to discriminate between acidic (pH 4), weakly acidic (4 pH 7) and alkaline (pH 7) GER episodes. In infants, pH-impedance represents a valuable diagnostic tool, as in this age group, GER episodes are more likely to be weakly acidic and/or alkaline, even in the absence of anti-secretory treatment. It has been estimated that almost 45% of infants diagnosed with GERD by MII pH-impedance would have had normal pH-metry [11]. However, in children no pH impedance parameter appears to correlate with the presence of esophagitis.

In adults, MII pH impedance monitoring enables the detection of non-acidic GER episodes that might be responsible of refractory symptoms during treatment. Furthermore, it can discriminate between “true” non-erosive reflux disease (NERD) patients (symptomatic patients with normal endoscopy and pathological esophageal acid exposure), patients with esophageal hypersensitivity (symptomatic patients with normal endoscopy, normal esophageal acid exposure and with positive symptom-reflux association probability) and functional heartburn patients (symptomatic patients with normal endoscopy, normal esophageal acid exposure and with negative symptom-reflux association probability). The identification of these clinical entities within the “GERD spectrum” is fundamental, as it directly influences the choice of treatment. In children, since symptoms are non-specific and reported by proxy, the symptom-reflux association probability is unreliable and the distinction between NERD, esophageal hypersensitivity and functional heartburn is not recommended [11]. As in adults, the role of new pH-impedance parameters, such as baseline impedance, is still matter of discussion. The measurement of baseline impedance was initially suggested to be useful in predicting the integrity of the esophageal mucosa and therefore in paving the way of selecting the treatment of infants and children with suspected esophageal mucosal injury [52,55]. However, this early hypothesis has not been consistently validated by further studies suggesting that baseline impedance might merely mirror the phenomena occurring either within the esophageal lumen, such as the acid reflux, or within the esophageal wall, such as the strength and coordination of esophageal peristalsis [56,57].

### 4.6. Manometry

Esophageal manometry is not indicated in the diagnostic algorithm of adult or pediatric GERD. In adults, the main goal of manometry is guiding the correct positioning of pH-impedance probes [55]. However, manometry might be helpful in excluding esophageal motor disorders (i.e., achalasia and esophageal spasm) and confirming the clinical suspicion of rumination syndrome, thus discriminating it from GERD. Consequently, although manometric studies are not routinely recommended in the management of GERD patients, particularly in children where the insertion of the probe might require sedation or general anesthesia, it is widely agreed that, when possible, esophageal manometry can be performed before considering antireflux surgery.

## 5. Treatment

The therapy of pediatric GERD is based on a combination of conservative measures (i.e., lifestyle and dietary modifications), pharmacological and, rarely, surgical treatment. As stated above, the proper state-of the-art approach relies on the correct diagnosis and evaluation of GERD patients. From a clinical standpoint, it is useful to distinguish between infants/young children and older children/adolescent GERD management, since the clinical presentation, the choice of the therapy and the response to treatment significantly differs between the two groups. Below, we will review the current evidence-based approach in infant GERD and we will then briefly discuss the approach to older children/adolescents complaining of typical GERD.

### 5.1. Non-Pharmacological GERD Management in Infants/Young Children

Conservative management is the current first line approach in infantile GERD. It ranges from feeding and posture modifications to modifying maternal diet in breast-fed infants. As previously discussed, in this setting, it is crucial to distinguish, “true” GERD and other clinical conditions that can resemble GERD, with CMPA being the most frequent.

Where there is clinical suspicion of GERD and the patient presents with alarm symptoms (see the list in paragraph 4.1), he should not be treated, but investigated accordingly (see above). On the contrary, in the absence of alarm features, patients should be treated conservatively by modifying feeds and posture. The feeding management strategy has been shown to represent an effective approach in the otherwise healthy infants with both GER and GERD. It involves modifying feeding frequency and volume, ensuring the intake of feed per kilogram of weight is appropriate. There is some evidence for the efficacy of feed thickeners on reducing visible regurgitation [58,59]. However, actual GER episodes are probably not reduced [58] and concerns have been raised about the putative association between food thickeners and necrotizing enterocolitis in preterm infants [60,61]. The Food and Drug Administration (FDA) currently discourages their use in infants born before 37 weeks of gestation. Patients with GER and GERD may also benefit from changing body position, by keeping them upright or even in the prone position, especially in the post-prandial period [62,63,64]. However, due to the increased incidence of sudden infant death syndrome (SIDS), it is not recommended to advise prone positioning for GERD during sleep [11], while the left lateral position appear a suitable alternative for the postural management of infant GERD.

If this first-line management fails to improve symptoms, current pediatric guidelines advise for a 2–4 weeks trial with cow’s milk protein free diet, by either excluding milk from maternal diet in breastfed infants or by using hydrolyzed formula in non-breast fed infants. This is the consequence of the fact that CMPA can resemble GERD and should always be considered as a possible differential diagnosis, particularly in patients with personal or familial history of atopy. Of note, this recommendation does not apply to the subset of GER patients, the so-called “happy spitters”.

### 5.2. Pharmacological GERD Management in Infants/Young Children

The pharmacological treatment of GERD encompasses anti-secretory and prokinetic drugs. In infants, however, the use of these agents must be reserved for patients with objectively assessed GERD and increased esophageal acid exposure.

Anti-secretory drugs are the backbone therapy for GERD patients. Both histamine-2 receptor antagonists (H_2_RAs), and proton pump inhibitors (PPIs) are approved for clinical use in the pediatric population, while, in infants aged 1 year, no proton pump inhibitor formulation is approved, at present. H_2_RAs are superior to placebo in healing erosive esophagitis in pediatric populations [65]. Nonetheless, unlike PPIs, H_2_RAs exhibit tachyphylaxis and tolerance and should not be considered in the long-term management of GERD.

PPIs have been shown to heal erosive esophagitis more effectively and rapidly than H_2_RAs and their chronic use is not associated with increasing tolerance [66]. According to current guidelines, PPIs should be prescribed at the lowest effective dose, once daily. As discussed elsewhere, abuse and inappropriate prescription of PPIs is a concerning issue, since it is associated with worrisome side effects, like increased lower respiratory infections, particularly in infants [67,68,69]. Furthermore, long-term PPIs therapy (2.5 years) has been associated with reduced bone mineralization, induced moderate hypergastrinemia and the development of enterochromaffin-like (ECL) cell hyperplasia in up to 50% of children [70]. Lastly, acid suppression itself is a recognized risk factor for community-acquired pneumonia, gastroenteritis, candidemia and necrotizing enterocolitis in preterm infants [71,72,73,74,75,76]. In addition, antacid drugs should not be considered in the management of infant GERD, as some absorbable components may induce side effects following chronic use (milk-alkali syndrome, aluminum toxicity, renal failure and hypercalcemia) [77,78,79]. Hence, the use of chronic acid suppression should always be weighed against the non-negligible risks of long-term therapy.

**Figure 1:**
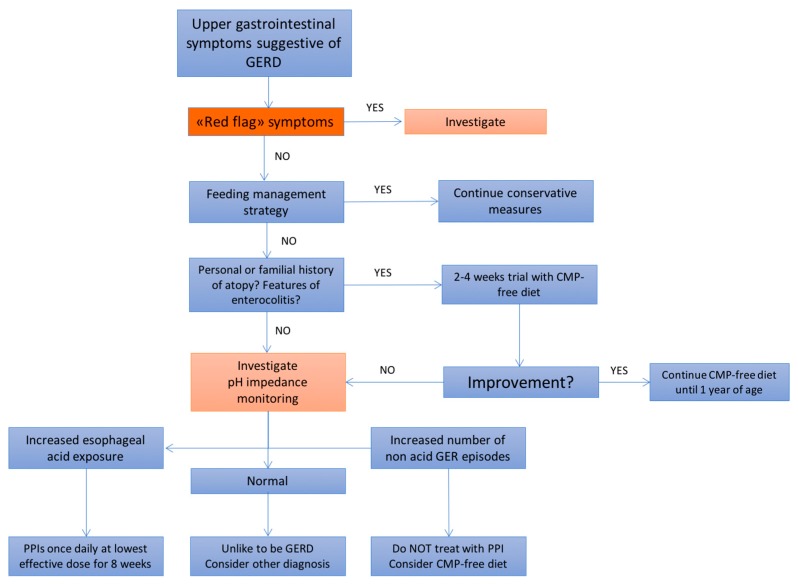
Flowchart of the clinical management of GERD in infants [11]. GERD: gastroesophageal reflux disease; GER: gastroesophageal reflux; CMP: Cow’s milk protein; PPIs: proton pump inhibitors.

The rationale of using prokinetic agents in GERD therapy relies on the evidence that these agents, by increasing gastric emptying rates, might reduce transient lower esophageal sphincter relaxation. However, all these therapeutic agents are associated with significant side effects, including extrapyramidal reactions and heart dysrhythmia; hence their use is currently not recommended [80,81]. Figure 1 summarizes the clinical management of GERD in infants according to current guidelines [11].

### 5.3. Older Children/Adolescent GERD

**Figure 2:**
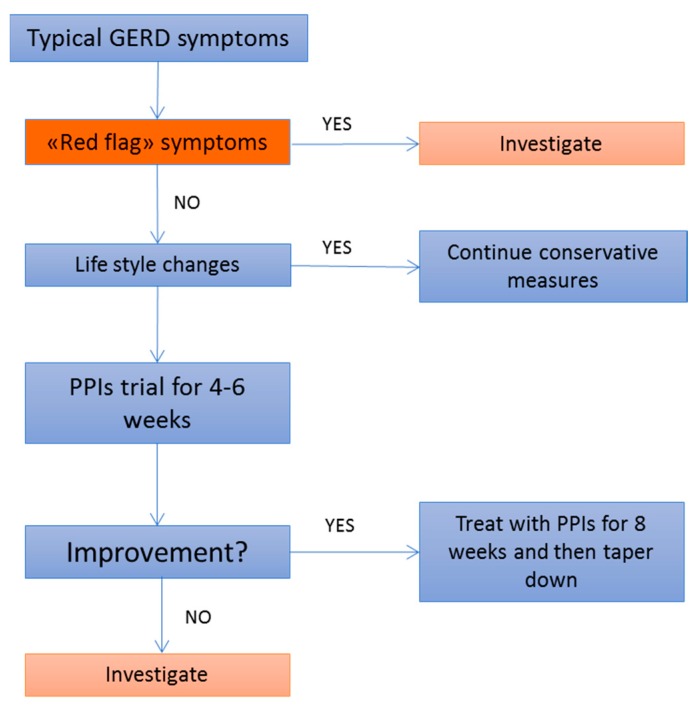
Flowchart of the clinical management of GERD in older children and adolescents [11]. PPIs: proton pump inhibitors.

The clinical management of typical GERD symptoms in older children and adolescents does not differ from that of their adult counterparts and it encompasses lifestyle modifications alongside with pharmacological treatment. The advised lifestyle changes for adolescents complaining of GERD resemble the adult recommendations, ranging from losing weight in overweight/obese adolescents to change of voluptuary habits, including cessation of smoking and alcohol avoidance. As in adults, dietary and positioning modifications can be advised in the conservative management of GERD in older children/adolescents. The use of caffeine, alcohol and acidic/spicy foods should be discouraged as well, as they are potentially able to trigger GERD symptoms. In addition, eating small, frequent meals, elevating the head of the bed and avoiding large meals before bedtime are first-line measures than can be easily applied in GERD management. As discussed before, in older children/adolescents, GERD tends to present with more typical symptoms, including heartburn and regurgitation, and the symptom-reporting is more reliable strengthening the likelihood of a clinical-based diagnosis of GERD. For these reasons, in absence of “red flag” symptoms, a PPI trial for 4–6 weeks can represent a reasonable first-line pharmacological approach in older children/adolescents complaining of typical GERD symptoms, alongside with lifestyle modifications (Figure 2). Refractory patients and patients complaining of atypical GERD symptoms, on the other hand, should be referred for further investigations [11].

### 5.4. Indication for Surgical Treatment

The indications for surgical treatment still represent a “grey area” in the management of pediatric GERD, as most of the literature on this topic is limited to retrospective series. According to current guidelines, anti-reflux surgery should be reserved for severe relapsing cases and for patients at high risk of long-term GERD complications [11]. Children, in which GERD is associated with respiratory comorbidities, including asthma and aspiration pneumonia, should be considered for surgery, although there is a lack of clinical evidence supporting this [11]. The failure of anti-secretory therapy does not represent an indication to surgery per se. On the contrary, the lack of response to pharmacological treatment should warn the clinician and should lead to careful reassessment of the patient, questioning GERD as underlying cause of the symptoms. Other more infrequent clinical conditions, such as eosinophilic esophagitis, cycling vomiting syndrome, gastroparesis and rumination syndrome should be ruled out by second and third level diagnostic tests (contrast studies, manometry), before considering further treatment. The careful selection of patients is a key process in children, since nearly 20% of patients require a redo of the fundoplication and the overall recurrence rate of symptoms varies between 5% and 15% in different studies [82]. Worse outcomes after Nissen’s fundoplication in children [79,80], compared to adults, has led to “uncertainty” whether the benefits of fundoplication in children outweigh the harm of long-term PPI use [71].

## 6. Conclusions

Despite the high prevalence of the disorder, GERD still represents a challenge, even for the experienced clinician. In pediatric populations, this is particularly evident due to the multifaceted clinical presentation and the frequent occurrence of regurgitation in “well infants”. There have been many efforts thus far to appropriately select patients for investigation and treatment and current guidelines represent the state-of-the-art approach to patients with suspected GERD. The recommended approach to adolescents complaining of typical GERD is similar to that of adults, encompassing lifestyle changes and a four-week trial with PPIs. In children aged less than two years, conservative measures (i.e., lifestyle and diet modifications) represent the first-line therapeutic strategy. Furthermore, due to the frequent overlap between GERD and CMPA, it is conceivable to advise a time limited trial of dietary restriction, especially in the presence of personal or family history of atopy. If non-pharmacological approaches fail, in the opinion of the authors, patients should be investigated by pH-impedance monitoring rather than be treated “ex iuvantibus”. The rationale for this strategy relies on the fact that GER in infants is often only mild acidic and/or alkaline, leading to inappropriate prescription and PPI abuse in this population. Acid-suppressive therapy is the cornerstone for the treatment of GERD in both adults and children. Although acid-suppressive drugs show a good profile of safety and tolerability, there is a real risk of adverse events that must be taken into account, particularly in long-term treatments. Surgical therapy should be reserved for chronic and relapsing cases and for patients at high risk of GERD complications. The rates of failure of fundoplication appear to be higher in children, but this might also reflect the poor selection of the patients and the still vague and controversial indications for surgical treatment.

## Figures and Tables

**Table 1:** Gastro-esophageal reflux disease (GERD)—Age related symptoms [10,11,12].

Age of the Patients	GERD Symptoms
Children 2 years of age	Regurgitation and vomiting
	Irritability with feeds and in postprandial period
	Back arching
	Crying
	Food refusal
	Cough
	Apnea
Children 3–17 years of age	Regurgitation and vomiting
	Heartburn
	Nausea
	Epigastric pain/stomachache
	Cough and wheezing

## Abbreviations

GER	Gastro-Esophageal Reflux
GERD	Gastro-Esophageal Reflux Disease
CMPA	Cow’s Milk Protein Allergy
GI	Gastro-Intestinal
EA	Esophageal Atresia
